# “Methods in Research and Development of Biomedical Devices”: the World Scientific Publishing Co. Pte. Ltd, 2013

**DOI:** 10.1186/1475-925X-13-121

**Published:** 2014-08-26

**Authors:** Robert Koprowski

**Affiliations:** Department of Biomedical Computer Systems, University of Silesia, Faculty of Computer Science and Materials Science, Institute of Computer Science, ul. Będzińska 39, Sosnowiec, 41-200 Poland

**Keywords:** Biomedical devices, Biomedical technologies, Image processing, Nasal drug delivery, Micropump, Stent, Treatment of aneurysms

## Abstract

This article is a review of the book “Methods in Research and Development of Biomedical Devices” (ISBN: 9789814434997, 98USD/65GBP, 177 pages) edited by Kelvin K L Wong, Jiyuan Tu, Zhonghua Sun and Don W Dissanayake published by the World Scientific Publishing Co. Pte. Ltd. in 2013. The content of the book and its importance for biomedical engineering have been discussed in this invited review.

## Book details

Title: “Methods in Research and Development of Biomedical Devices”,

ISBN: 9789814434997,

Price: 98USD/65GBP,

Number of pages: 177,

Edited by: Kelvin K L Wong, Jiyuan Tu, Zhonghua Sun and Don W Dissanayake,

Published by: the World Scientific Publishing Co. Pte. Ltd. in 2013.

### Book review

The book titled "Methods in Research and Development of Biomedical Devices" presented for a review was written by Dr. Kelvin KL Wong, Prof. Jiyuan Tu, Prof. Zhonghua Sun and Dr. Don W Dissanayake. The book is published in hardcover and consists of 177 pages divided into 9 chapters, bibliography (239 items), answers to the questions contained in the individual chapters (an average of about 5 questions per chapter) and index. The description of the contents of each chapter is preceded by a foreword written by the eminent Professor Dhanjoo Ghista (among other things, a member of the editorial board of the journal BioMedical Engineering OnLine) and further there is a preface and acknowledgments. Individual chapters raise issues such as: overview of biomedical technologies, conceptualisation and medical image-based modelling, medical imaging and visualisation, treatment of aneurysms, endovascular stent grafts, nasal drug delivery, biomedical MEMS micropump and engineering and production management for biomedical devices. The book contains numerous gray-level illustrations with the dominance of interesting cases of 3D reconstruction. The organization of the book, the selection of topics of individual chapters and their structure are intuitive. Each chapter also ends with a few questions and the answers are found at the end of the book.

The book discusses the issues of planning, prototyping and implementation of selected tools and instruments used in medicine. In each chapter the number of mathematical formulas is limited to a minimum, thus increasing the substantive scope by showing practical examples from the field of bioengineering. The examples that dominate here relate to stents, treatment of aneurysms, imaging and 3D reconstruction as well as nasal drug delivery. The descriptions of particular issues made by the authors refer to practical cases and solutions. This is undoubtedly a very big advantage of this monograph.

### Specific content

The first chapter presents the basic problems of designing biomedical devices. In this short chapter, a block diagram showing the various stages of designing biomedical devices has been presented. In addition, the key issues related to measurement, prototyping, testing and implementation of biomedical devices have been discussed.

The second chapter provides an overview of biomedical technologies. The issues of stents, micropumps and drug delivery devices have been highlighted in a special manner.

The next chapter concerns the development of the concept of implementation and modelling of biomedical devices discussed in the previous chapter. Particular attention is paid here to computer simulation, biomedical imaging and reconstruction of images as well as prototyping with the use of 3D printing.

The fourth chapter focuses on biomedical imaging. Among others, the issues related to computed tomography imaging or endoscopy are discussed here. A significant part of the chapter is devoted to issues of three-dimensional image analysis and processing. The impact of the binarization threshold selection on the obtained reconstruction results is discussed in a very precise way.

The next chapter addresses the methodology for the treatment of aneurysms. Minimally invasive methods, technical limitations of treatment and three-dimensional computer reconstruction methods used in such cases are presented here.

The sixth chapter is devoted to endovascular stent grafts. A significant part of the description herein refers to the technical description of this type of stent. Considerations concerning the behaviour of the stent also in dynamic states of blood pulsation are presented. The issues of the next two chapters (seventh and eighth), namely nasal drug delivery and biomedical MEMS micropump, are explored in an equally curious and interesting way. They contain an accurate description of computer modelling, theoretical analysis (in the case of micropumps) and estimation of the parameters received.

The last ninth chapter focuses on production issues related to biomedical devices. This chapter provides a summary of the issues presented earlier. The authors pay particular attention here to design, prototyping and protection of biomedical devices and their marketing. This last chapter will be especially important for readers seeking research funding from external sources (grants, international projects, etc.).

### Strengths of the book

The book covers the major problems encountered during the design, simulation and prototyping of biomedical devices. It perfectly fills the gap between the theoretical issues related to biomedical devices and specific electronic devices and information technology programs (mainly designed for image analysis and processing).

There exist very good books in the field of analysis and processing of 2D [1] and 3D [2] biomedical images. There are also well-known books in the field of biomedical materials science [3] or medical descriptions of various diseases as well as descriptions of limitations in the construction of biomedical devices [4]. However, such a good interdisciplinary combination of issues related to computer science, electronics and medicine, and mainly computer science and medicine, has been shown so far in very few books (e.g. [5]). For example, chapter 6 - dedicated to the review of the device and technical evolution of stent grafts or chapter 8 - computer modelling and analysis of MEMS micropumps of biomedical applications. This is extremely important because it shows the reader the full path which in practice should be covered from the idea to implementation of a biomedical device prototype. This prevents misunderstandings arising at the intersection of physician-engineer cooperation, especially in terms of the resolution and accuracy of the results obtained as well as broad technical possibilities.

In comparison to the afore-mentioned available and known publications, the reviewed book is an extremely valuable interdisciplinary addition to practical knowledge in the field of treatment of aneurysms, stent grafts, nasal drug delivery and biomedical micropump. This range covers a significant part of the book - 72 pages. Therefore it is a very important book for both engineers and students, PhD students or physicians who want to expand their range of knowledge in these areas.

Numerous images (more than 80) and a small number of mathematical formulas (less than 10) mean that the group of readers may also include people not professionally connected with the presented issues. This is an extremely important feature that contributes to the popularity and distribution of books, both in academic institutions and those implementing new products and biomedical devices.

The book can also be used in teaching owing to the questions provided at the end of each chapter (the answers are given at the end of the book). The book can also be used in courses devoted to the issue of "medical devices" in the afore-mentioned areas of knowledge. In this respect, it is at the same time the greatest authors’ contribution to the current state of knowledge.

### Weaknesses of the book

A brief book on a collection of very large topics has its strengths and weaknesses. It can introduce the reader to a range of topics, but many readers would need a deeper discussion than can be offered in brief chapters.

The authors wrote that they "hope that this book can be of use to biomedical engineers, medical doctors, radiologists and any other professionals related to the research and development of medical devices for health care". In the area "development of medical devices" readers can expect a more thorough analysis of the problem both in terms of theory, for example, different definitions, classifications and requirements for biomedical devices, and practice, i.e. a greater number of examples and their more comprehensive descriptions. For example, "Overview of Biomedical Technologies" has only 9 pages and "Medical Imaging and Visualization" has only 18 pages. Similarly, some subchapters, such as "Computed Tomography", consist of 1 page only. The consequence of trying to fit a large substantive scope on a relatively small number of pages is the text division into short sections and subsections. For example, there are 2 or 3 subsections on one page.

Similarly, readers may require more information in terms of teaching aspects of the book. The authors wrote in the Preface that "it is also an ideal postgraduate teaching material and may also be promoted by lecturers as a textbook and manual guide". The authors may also consider expanding the number of questions provided at the end of chapters to 10 or 20 in future editions of the book. In my opinion, it will be more useful in courses and teaching in the field of biomedical devices. A textbook should also contain more rudiments of relevant problems, design tasks for students and links to pertinent materials and teaching aids. When considering the educational aspects of the book, its price, which seems to be too high for students, is also vital.

The authors also wrote that "this book presents a roadmap for applying the stages in conceptualisation, evaluation and testing of new medical technologies in a systematic order of approach, leading to solutions for medical problems with a well-deserved safety limit". The "roadmap" mentioned by the authors is partially presented. Only the sections "Overview of Biomedical Technologies" are "Engineering and Production Management for Biomedical Devices" are helpful here. The other sections of the book show only examples. They lack a direct reference to the "roadmap" which the authors mentioned.

Probably, many readers will need more explanation the reasons and link for such a choice of subjects of individual chapters (treatment of aneurysms, stent grafts, nasal drug delivery and biomedical micropump). A minor shortcoming is also too small and cursory reference to the classification, standardization and regulation of biomedical devices in different countries of the world (e.g., in the discussed practical examples).

In the sections directly related to practical examples of biomedical devices, it is difficult to find justification for the used simplifications of material groups. There are numerous 3D images whereas readers may be interested in their accuracy and repeatability for various types of pathology.

Finally it should be noted that in the book I have noticed a few minor typos and grammatical and nomenclature errors. Some of the descriptions shown in the images are not legible (e.g. scale in Figure two point two). Some image descriptions mention colours whereas the image is in gray levels (e.g. Figure three point nine - blue). My minor comments also relate to differences in quality between various graphs such as between Figure six point thirteen and Figure six point fourteen or Figure six point sixteen.

However, for what it attempts to do, the book is interesting and useful, and I have a high opinion of this monograph.

## Conclusion

This is an interesting book in the field of biomedical devices, containing many practical examples. The individual chapters are presented in a clear and lucid manner, which undoubtedly expands the group of potential readers to a great extent.
